# Expanded Hemodialysis with Theranova 500 Improves Dialysis Adequacy and Blunts Inflammation: A 24-Week Quasi-Randomized Trial

**DOI:** 10.3390/jcm14248853

**Published:** 2025-12-14

**Authors:** Nomy Levin Iaina, Elena Rotshild, Sharon Mini Goldberg, Pazit Beckerman

**Affiliations:** 1 Department of Nephrology and Hypertension, Barzilai University Medical Center, Ashkelon 7830604, Israel; elenar@bmc.gov.il; 2 School of Medicine, Faculty of Health Sciences, Ben Gurion University of the Negev, Beer Sheva 8410501, Israel; 3 Institute of Nephrology and Hypertension, Sheba Medical Center, Ramat Gan 5265601, Israel; sharon.mini@sheba.health.gov.il (S.M.G.); pazit.beckerman@sheba.health.gov.il (P.B.)

**Keywords:** dialysis adequacy, hemodialysis, inflammation, medium cut-off membrane, quality of life

## Abstract

**Background/Objectives**: Uremic middle molecules contribute to chronic inflammation and symptom burden in hemodialysis patients. The Theranova 500 medium-cutoff (MCO) dialyzer enhances clearance of larger uremic toxins and may offer clinical advantages. We hypothesized that Theranova 500 would improve dialysis adequacy (spKt/V), attenuate inflammation (CRP), and provide targeted improvement in symptom burden compared with the high-flux Revaclear 500. **Methods**: We conducted an open-label, prospective, quasi-randomized controlled trial including forty prevalent adult hemodialysis patients from two centers in Israel (Barzilai and Sheba Medical Centers). Patients were sequentially allocated 1:1 to Theranova 500 or Revaclear 500. Demographic, laboratory, and patient-reported outcomes (KDQOL-SF, Dialysis Symptom Index) were assessed at baseline and week 24. Within-group changes were analyzed using paired tests, and between-group differences using ANCOVA adjusted for baseline values. Safety monitoring included adverse events, dialyzer reactions, hospitalizations, and mortality. **Results**: Theranova 500 significantly increased mean spKt/V (1.24 ± 0.33 to 1.40 ± 0.36; *p* = 0.025), while Revaclear showed no significant change. CRP remained stable in the Theranova group but rose nearly threefold in the Revaclear group by week 24 (*p* < 0.001). Albumin, dry weight, anemia and mineral bone parameters remained stable in both groups. Total cholesterol increased modestly in the Theranova arm without nutritional compromise. Symptom-level analysis showed improvement in irritability, restless leg, dry skin, chest pain, and diarrhea with Theranova, whereas global KDQOL-SF domain scores improved similarly in both groups. No non-serious adverse events, hypersensitivity reactions, or dialyzer-related intolerance was observed. Hospitalizations (n = 8 per group) and mortality (two per group) were identical. **Conclusions**: Over 24 weeks, Theranova 500 MCO dialyzer improved dialysis adequacy and prevented the rise in inflammatory markers seen with Revaclear without compromising nutrition or safety. Targeted improvement in specific uremic symptoms suggests potential clinical benefit beyond small-molecule clearance. These findings support the safety and clinical utility of expanded hemodialysis and highlight the need for larger, fully randomized trials to validate these results.

## 1. Introduction

Hemodialysis (HD) remains the cornerstone of renal replacement therapy for End-Stage Renal Disease (ESRD), yet patients on chronic HD continue to face markedly higher morbidity and mortality than the general population [1,2]. In addition to the longstanding risks of cardiovascular and infectious complications, many patients experience burdensome symptoms, including fatigue, generalized weakness, and pruritus, that impair daily function and quality of life.

A key contributor to these adverse outcomes is the retention of “middle-molecule” uremic toxins, typically defined as solutes between 15 and 45 kDa. Conventional low-flux membranes are essentially unable to remove these solutes, and even high-flux (HF) dialyzers achieve only partial clearance [2,3]. Accumulation of larger middle molecules, pro-inflammatory cytokines such as interleukin-6 (IL-6) and tumor necrosis factor-α (TNF-α), and free light chains (FLCs) promotes chronic inflammation [4], endothelial dysfunction and vascular calcification [5], and protein-energy wasting, all of which exacerbate symptom burden and heighten cardiovascular risk [6].

To address this gap, medium-cutoff (MCO) membranes, such as Theranova 500 (Baxter International Inc. Deerfield Illinois, USA), have been engineered with a narrower pore size distribution and enhanced internal filtration (“internal hemodiafiltration”), which enable efficient removal of solutes up to ~45–56 kDa while limiting albumin loss to clinically acceptable levels [7,8,9]. In vitro studies and small pilot trials confirm that Theranova effectively clears larger middle molecules (e.g., λ light chains) with only minimal, non-clinically significant declines in serum albumin.

Although MCO membranes have been adopted in dialysis centers worldwide and real-world registries, including the Colombian COREXH cohort of nearly 1000 patients, demonstrate stable albumin levels, mortality of 8.5 deaths/100 patient-years, and hospitalization rates of 0.79 events/100 patient-years, comparable to HF [10], data on their effects on dialysis adequacy, inflammation, laboratory parameters, symptom relief, and patient-reported outcomes remain limited.

The aim of this study was to compare the Theranova 500 (MCO) dialyzer with the Revaclear 500 (HF) dialyzer ((Baxter International Inc. Deerfield Illinois, USA), aiming to determine whether expanded middle-molecule clearance translates into superior dialysis efficacy, more favorable changes in inflammatory biomarkers and routine laboratory tests, and meaningful improvements in symptom burden, quality of life, and overall clinical outcomes. The primary objective was to evaluate whether treatment with the Theranova 500 dialyzer improves dialysis adequacy, assessed by spKt/V, and systemic inflammatory status, assessed by C-reactive protein (CRP), compared with the Revaclear 500 dialyzer over a 24-week period. The secondary objectives were to assess changes in nutritional and metabolic parameters, including serum albumin, lipid profile, and dry weight, to evaluate effects on anemia and mineral bone markers, to compare changes in patient-reported outcomes using validated tools (KDQOL-SF and Dialysis Symptom Index) and to assess safety outcomes, including the rate of hospitalizations and all-cause mortality.

## 2. Materials and Methods

### 2.1. Study Design

This was a 24-week, prospective, open-label, quasi-randomized controlled trial conducted in two hemodialysis units in Israel—Barzilai University Medical Center (Southern District, Ashkelon) and Sheba Medical Center (Central District, Ramat Gan)—between February 2022 and March 2023. The study adhered to the principles of the Declaration of Helsinki and received approval from the Institutional Helsinki Committees of Barzilai and Sheba Medical Centers (approval no. 0098-21-BRZ). All participants provided written informed consent prior to enrollment. The study was not registered in a clinical trial registry as both dialyzers are routinely used clinical devices and the design involved quasi-random allocation rather than a formal randomized clinical trial. This was an observational study based on conventional treatment, rather than a new clinical intervention.

### 2.2. Inclusion and Exclusion Criteria

Patients were eligible if they were aged ≥18 years at the time of consent, of either sex, had been receiving chronic HD for ≥90 days via an arteriovenous fistula (AVF) or graft (AVG), demonstrated dialysis adequacy of spKt/V > 1.2, and could provide informed consent. Patients were excluded for any of the following: known hypersensitivity to study dialyzers, use of a temporary dialysis catheter, planned renal transplantation within 12 months, hospitalization within 3 months prior to enrollment, active malignancy or ongoing sepsis, or intensive care unit admission at screening. Subjects were withdrawn upon patient request, development of hypersensitivity reactions to the study dialyzer or at the discretion of the treating nephrologist for any medical reason.

### 2.3. Patients Allocation and Study Groups

Forty eligible patients were sequentially allocated in a 1:1 ratio to Theranova 500 medium-cutoff (MCO) dialyzer or Revaclear 500 high-flux (HF) dialyzer treatments. Allocation was performed sequentially according to enrollment order: the first enrolled patient was assigned to the Theranova group, the second to the Revaclear group, and so on. Thus, the study design represents a quasi-randomized allocation process. Importantly, investigators had no role in determining treatment assignment. Allocation was performed by study coordinators and communicated to the investigator, who instructed the appropriate dialysis setup. Patients in the Theranova group had their dialyzers switched from Revaclear 500 to Theranova 500, while those in the Revaclear group continued with their existing treatment. Key properties of the Theranova 500 (MCO) and Revaclear 500 (HF) dialyzers, including membrane composition, effective surface area, ultrafiltration coefficient, and molecular-weight cutoff, are detailed in Table 1.

### 2.4. Study Procedures

Baseline demographic and clinical data, including age, HD vintage, etiology of ESRD, vascular access type and medical history were recorded. Dialysis parameters (sessions per week, session duration, dialyzer type, blood flow rate, dialysate flow rate, ultrafiltration volume, and dry weight) were documented at baseline, week 12, and week 24.

Laboratory assessments, comprised complete blood count, comprehensive metabolic panel, parathyroid hormone, iron stores, serum albumin, lipid profile, C-reactive protein, and HbA1c in diabetic patients, were recorded at baseline, week 12, and week 24. Dialysis adequacy was measured by single-pool Kt/V (spKt/V).

Quality of life and symptom burden were evaluated using two validated Hebrew questionnaires: the Kidney Disease Quality of Life Short Form (KDQoL-SF) and the Dialysis Symptom Index (DSI). Questionnaires scores at 24 week compared to baseline. Scoring of the three KDQOL-SF domains (Physical Component Summary, Mental Component Summary and Kidney Disease Component Summary) was performed according to the official RAND KDQOL-SF™ v1.3 manual [11].

The number of hospitalizations and mortality rate during the 24-week treatment period was documented. Safety monitoring also included documentation of any adverse events, including dialyzer-related reactions or intolerance during hemodialysis sessions.

### 2.5. Statistical Analysis

Continuous variables were examined for normality using both visual inspection of histograms and the Shapiro–Wilk test. Normally distributed variables are presented as mean ± SD, whereas skewed variables are summarized as median (interquartile range). Between-group comparisons at each time point were performed using independent-samples *t*-tests for normally distributed variables and the Wilcoxon rank-sum test for non-normally distributed variables. Within-group longitudinal changes (baseline vs. week 12 vs. week 24) were assessed using paired *t*-tests or Wilcoxon signed-rank tests, as appropriate. To account for baseline variability in key parameters, analysis of covariance (ANCOVA) was used for selected outcomes, with baseline values entered as covariates. Categorical variables were compared using χ^2^ or Fisher’s exact test when expected cell counts were small. Correlations between continuous parameters were assessed using Pearson or Spearman correlation coefficients according to distribution. No correction for multiple comparisons was applied, given the exploratory nature of the secondary outcomes. Missing data were rare (<5%) and were handled by complete-case analysis without imputation. All tests were two-sided, and statistical significance was defined as *p* < 0.05. Analyses were conducted using SPSS Statistics (version 27; IBM Corp., Armonk, NY, USA).

## 3. Results

**Figure 1:**
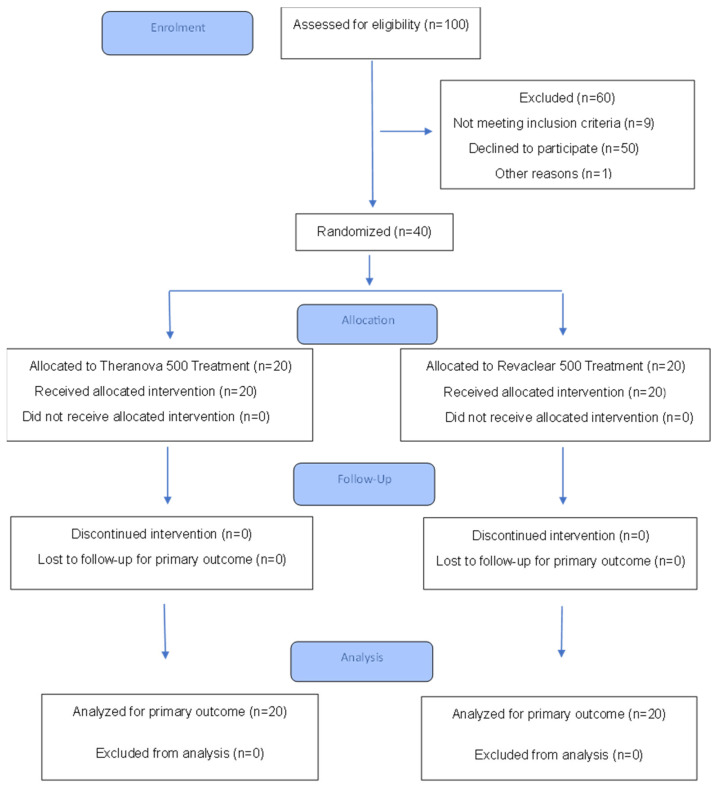
CONSORT 2025 Flow Diagram. Flow diagram of the progress through the phases of a randomized trial of two groups (that is, enrolment, intervention allocation, follow-up, and data analysis).

A total of 40 patients participated in this study, 20 in the Theranova group and 20 in the Revaclear group. Allocation was balanced as described in the Methods section. The flow of participants through the study is summarized in Figure 1. Baseline characteristics data is presented in Table 2. No statistically significant differences were observed between the groups in terms of age, dialysis vintage, ESRD cause or vascular access type. Although no statistically significant differences were detected, the Revaclear group exhibited numerically higher rates of cardiovascular comorbidities (including diabetes, dyslipidemia, and atrial fibrillation), which may reflect a slightly higher baseline risk profile.

### 3.1. Dialysis Adequacy

No significant differences were found between the groups in spKt/V, dry weight, or UF volume at baseline, 12 weeks, and 24 weeks (Table 3). Baseline spKt/V was slightly lower in the Theranova group than in the Revaclear group (1.24 ± 0.33 vs. 1.39 ± 0.32), although this difference was not statistically significant.

**Figure 2:**
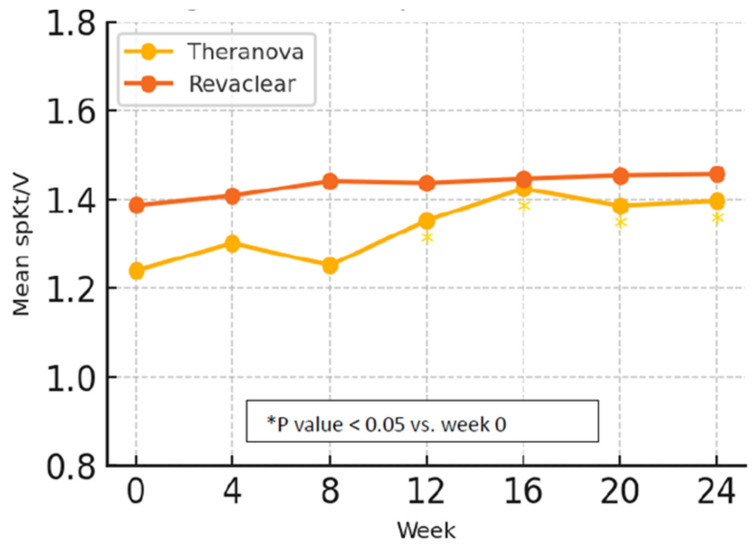
Mean spKt/V over 24 weeks. Mean spKt/V measured at weeks 0, 4, 8, 12, 16, 20, and 24 for patients treated with Theranova 500 and Revaclear 500. Data points represent group means; * mark time points with a significant within-group increase in spKt/V compared to week 0 (*p* < 0.05).

However, a statistically significant increase in spKt/V was observed from baseline to week 24 in the Theranova group (*p* = 0.025), starting from week 12, while no significant change was noted in the Revaclear group (Figure 2).

### 3.2. Anemia, Iron Indices and Mineral Metabolism

Hemoglobin and iron stores parameters are presented in Table 4. Hemoglobin levels were significantly higher in the Revaclear group in week 24 compared to the Theranova group (*p* < 0.05). No significant differences were observed between groups in ferritin and transferrin saturation. No significant differences were found in serum calcium, phosphate, or PTH levels between the two groups at any time point (Table 4).

### 3.3. Inflammation and Nutritional Parameters

**Figure 3:**
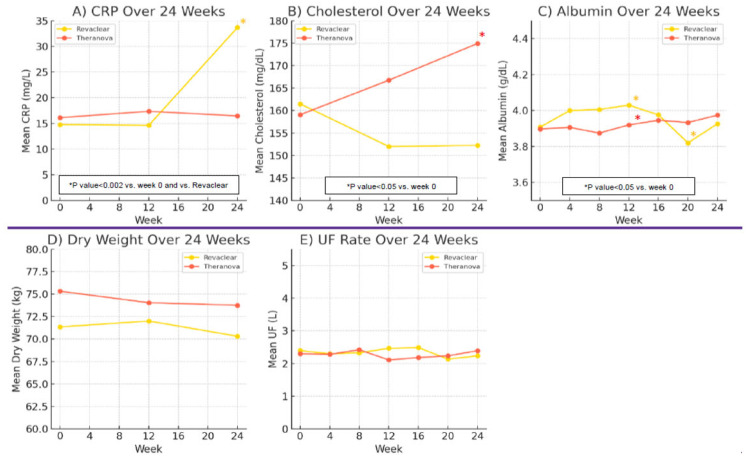
Inflammation and nutritional parameters over 24 weeks. (**A**): Mean C reactive protein (CRP) trajectories for Theranova 500 and Revaclear 500. (**B**): Mean total cholesterol trajectories for Theranova 500 and Revaclear 500. (**C**): Mean dry weight trends for Theranova 500 and Revaclear 500. (**D**): Mean serum albumin levels for Theranova 500 and Revaclear 500. (**E**): Mean ultrafiltration (UF) volume per session for Theranova 500 and Revaclear 500. All panels display group means at weeks 0, 12, and 24; * mark significant within-group changes versus week 0 (*p* < 0.05).

Mean CRP levels did not differ significantly between groups at baseline or week 12 but rose sharply in the Revaclear group by week 24 (*p* < 0.001 vs. week 0) and were significantly higher than the mean CRP levels in Theranova group (*p* = 0.002) (Figure 3A). In the Theranova group, mean CRP levels remained stable throughout the study. Total cholesterol levels rose significantly in the Theranova group by week 24 compared with baseline (*p* = 0.02), whereas no significant change was observed in the Revaclear group (Figure 3B). Serum albumin increased significantly from baseline to week 12 in both Revaclear (*p* = 0.001) and Theranova (*p* < 0.05), and they remained stable in Theranova and significantly decreased in the Revaclear group (<0.05) by week 24 (Figure 3C). There were no significant changes in dry weight and ultrafiltration rates during the study (Figure 3D,E).

### 3.4. Quality of Life Assessment

**Figure 4:**
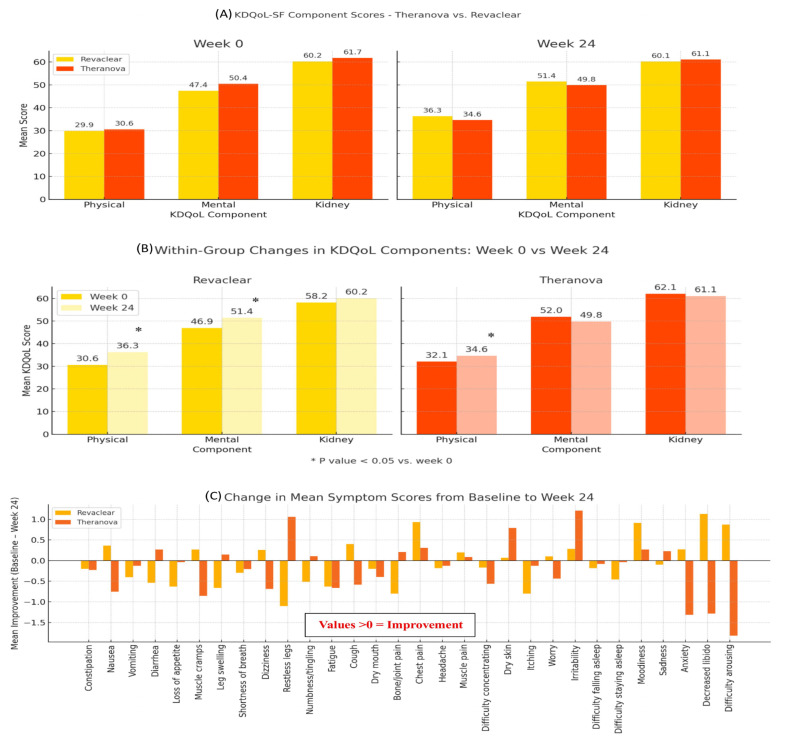
Quality of life and symptom outcomes. (**A**): Between-group comparison of KDQOL SF physical, mental and kidney disease component summary scores at weeks 0 and 24 for Theranova 500 and Revaclear 500. (**B**): Within-group mean change in each KDQOL SF domain from week 0 to 24; bars denote mean ± SD. (**C**): Change in Dialysis Symptom Index scores for Theranova 500 and Revaclear 500; positive values indicate symptom relief.

All items in the Kidney Disease Quality of Life Short Form (KDQoL-SF) and Dialysis Symptom Index (DSI) questionnaires were aggregated for statistical analysis, and comparisons were made between baseline and week 24. In both treatment arms, baseline and 24-week KDQOL-SF physical, mental, and kidney disease component scores were comparable (Figure 4A). Over 24 weeks, within-group paired analyses revealed that patients in the Revaclear group experienced statistically significant improvements in the physical and mental components (*p* = 0.010 and *p* = 0.030, respectively), whereas the kidney disease component remained unchanged (Figure 4B). In the Theranova arm, a significant increase was observed only in the physical component (*p* = 0.020), with no meaningful changes in mental or kidney components (Figure 4B).

The Theranova group showed greater improvement across more quality of life items, at 52 of 80 measures (65%) compared to 47 of 80 measures (58%) in the Revaclear group; however, the observed difference in improvement rates did not reach statistical significance.

No statistically significant improvement was observed between groups in the DSI questionnaire overall scores. Symptoms that demonstrated the largest relative improvement in the Theranova group included irritability, restless legs, dry skin, chest pain, and diarrhea. Conversely, several symptoms—such as pruritus, reduced appetite, sleep disturbances, and difficulty concentrating—showed minimal or no clinically meaningful change over the 24-week period (Figure 4C). In contrast, the Revaclear group’s top improvements were in decreased libido, difficulty arousing, moodiness, chest pain, and cough (Figure 4C). Despite these item-level differences, there were no statistically significant differences in the number of symptoms improved between groups.

### 3.5. Safety Monitoring, Hospitalizations and Mortality

No non-serious adverse events, hypersensitivity reactions, or dialyzer-related intolerance events were observed in either group during the study period. During the 24-week study period, a total of eight hospitalizations occurred in each treatment arm, with no between-group differences. All-cause mortality was also identical, with four deaths reported overall, two in each group, with no significant difference in survival.

## 4. Discussion

In this 24-week prospective quasi-randomized trial, we compared the effects of an MCO dialyzer (Theranova 500) versus a standard HF dialyzer (Revaclear 500) on dialysis adequacy, laboratory parameters, and patient-reported outcomes in chronic hemodialysis patients. We had hypothesized that Theranova 500 would improve dialysis adequacy (spKt/V), attenuate inflammation (CRP), and reduce patient symptom burden versus standard high-flux Revaclear 500. Direct middle-molecule markers such as β2-microglobulin, IL-6, and full free light chain profiles were not systematically measured in all participants. We performed random sampling of FLC clearance at baseline, week 12, and week 24, which consistently demonstrated effective removal with the MCO membrane. Given prior robust evidence confirming superior middle-molecule clearance with MCO dialyzers, we focused primarily on clinical, inflammatory, and patient-reported outcomes.

We found that over 24 weeks, Theranova 500 MCO dialyzer enhanced small-molecule clearance, increased spKt/V, and blunted inflammatory rise, without compromising nutrition or safety. Comparable global quality of life gains highlight that mechanistic benefits of MCO therapy may be most apparent in laboratory markers.

Although both groups had comparable baseline characteristics, the Theranova arm demonstrated a statistically significant increase in spKt/V, despite equivalent blood and dialysate flows, suggesting that expanded middle-molecule clearance and enhanced internal filtration translate into modestly improved small-molecule removal. By contrast, the Revaclear group’s spKt/V did not change significantly. Similar mechanistic studies have demonstrated improved reduction ratios for urea and β_2_ microglobulin with MCO membranes compared to high-flux dialyzers, without compromising fluid or electrolyte management [12,13]. Importantly, baseline spKt/V values were marginally lower in the Theranova group. This numerical imbalance may reflect random variation in a small sample and should be considered when interpreting the magnitude of within-group changes but could be partially accounted for by the greater relative improvement observed during the study. Larger studies with balanced baseline adequacy parameters are needed to confirm these findings.

Our trial provides evidence that expanded middle-molecule clearance can translate into incremental gains in small-molecule removal. Unlike most prior work, which has focused primarily on surrogate solute kinetics or broad quality of life domains, we paired these quantitative clearance data with detailed inflammatory, nutritional, and lipid biomarker trajectories, confirming that better solute removal does not come at the expense of albumin loss or malnutrition [14,15,16,17].

In our trial, hemoglobin levels and iron indices, as well as serum calcium, phosphate, and parathyroid hormone concentrations, remained essentially identical in the Theranova and Revaclear arms, indicating no impact of MCO versus HF membranes on anemia management or mineral bone parameters. These findings are in line with prior randomized studies showing comparable hemoglobin, ESA requirements, and iron status between MCO and conventional HF or HDF dialysis over 6–12 months [18,19,20]. They are also in line with multicenter trials reporting no significant changes in phosphate, calcium, or PTH levels when using MCO membranes in place of HF or online HDF modalities (18–20).

Our trial also provides key insights into the inflammatory and nutritional consequences of MCO dialysis. Whereas CRP rose nearly threefold in the Revaclear arm over 24 weeks, it remained essentially unchanged with Theranova, underscoring that expanded middle-molecule clearance may mitigate pro-inflammatory toxin accumulation without triggering compensatory inflammatory responses. This contrasts with conventional high-flux membranes, which may allow retention of larger cytokines and free light chains that perpetuate chronic inflammation. Importantly, the Revaclear cohort presented a numerically higher burden of cardiovascular comorbidities at baseline, although not statistically significant, which may have predisposed this group to a greater inflammatory response and could partially explain the divergent CRP trajectories. Given the small cohort size, multivariable adjustment for comorbidities was not statistically feasible; thus, residual confounding cannot be excluded. This potential residual confounding highlights the need to interpret the findings with caution and supports the importance of larger, fully randomized trials to confirm these observations. We confirmed that the small, transient albumin losses described in early MCO trials did not translate into sustained hypoalbuminemia or weight loss. Instead, both serum albumin and body weight remained stable in our Theranova cohort. Moreover, the observed increase in total cholesterol, often interpreted as a marker of improved caloric intake and anabolic status in dialysis patients, suggests that MCO dialysis may foster a more favorable metabolic milieu. Previous randomized trials and observational cohorts reported no sustained differences in IL 6, TNFα, or CRP between MCO and HF or HDF modalities, despite superior in vitro clearance of middle molecules [15,17,20].

Patient-reported outcomes provided complementary insights. Although global KDQOL-SF component scores improved similarly in both arms and between-group differences were not statistically significant, the symptom-level analysis revealed that 65% of individual QoL items improved with Theranova versus 58% with Revaclear. At the item level, the Theranova group showed the greatest improvement in irritability, restless legs, dry skin, chest pain, and diarrhea—symptoms often associated with middle-molecule accumulation—whereas other domains such as pruritus, sleep quality, and appetite showed limited change. However, there were no statistically significant differences between the groups in the number of symptoms improved, underscoring that the clinical impact of MCO therapy may be most evident at the individual symptom level, rather than in aggregate scores. These findings are consistent with prior RCTs and real-world analyses showing equivalent or modestly superior improvements in physical functioning, energy/fatigue, and post-dialysis recovery time with MCO membranes, with no consistent effects on pruritus or sleep quality [18,21].

Although CRP levels increased substantially in the Revaclear group, we showed that the incidence of hospital admissions and all-cause mortality did not differ between the Theranova and Revaclear arms, supporting the overall safety profile of expanded hemodialysis membranes. The small sample size and low event rates limit statistical power to detect modest differences in clinical outcomes, and therefore a potential relationship between inflammatory trajectories and subsequent hospitalization risk cannot be excluded. Our results mirror large adult cohorts and registry analyses, including both retrospective studies and pragmatic RCT post hoc analyses, that uniformly report no significant differences in hospitalization rates, lengths of stay, or survival when comparing MCO (Theranova) versus HF HD or HDF over 1–4 years of follow up [14,20,22,23,24]. Taken together, these data reinforce that the adoption of MCO dialyzers carries no incremental risk of adverse clinical outcomes in the maintenance HD population.

Strengths of this study include its systematic assessment of both laboratory and patient-reported outcomes and the use of validated tools (KDQOL SF and DSI) to capture symptom burden. Nonetheless, several limitations merit consideration. The study was not strictly randomized, as allocation followed a sequential alternating pattern rather than concealed randomization. This quasi-randomized approach carries an inherent risk of selection bias. Although no significant baseline differences were detected, patients in the Revaclear group tended to have more cardiovascular comorbidities, which could theoretically influence inflammatory trajectories or hospitalization risk. These limitations should temper causal interpretation, and confirmatory trials with fully randomized, concealed allocation are warranted.

In addition, the sample size may limit generalizability and statistical power to detect modest between-group differences, particularly in patient-reported endpoints. The open-label design could introduce reporting bias, and the 24-week follow-up may be insufficient to assess longer term effects on hospitalization, mortality, and nutritional status. The external validity of our findings is limited by the relatively small and clinically homogeneous study population, derived from two centers within a single healthcare system. As such, the results may not fully generalize to more heterogeneous hemodialysis populations with differing demographic, comorbidity, or treatment characteristics. Larger, multicenter trials including diverse patient groups are needed to confirm the applicability of these findings across broader clinical settings.

Finally, baseline symptom burden was not stratified, potentially diluting treatment effects in subgroups with the highest uremic symptom loads. Indeed, most published MCO versus HF or HDF trials share similar limitations, short duration (≤12 months), modest sample sizes, open-label designs, and low event rates.

In conclusion, Theranova 500 conferred a significant improvement in dialysis adequacy and maintained stable inflammatory and nutritional biomarkers over 24 weeks. Both MCO and HF dialyzers were safe, with no excess albumin loss, hospitalizations, or mortality differences. Symptom-level analyses suggest that MCO membranes may provide targeted relief for middle-molecule-associated symptoms.

This trial uniquely integrates rigorous solute-kinetic data with detailed inflammatory, nutritional, and global patient-reported outcomes, representing a balanced approach to toxin clearance and patient wellbeing. Larger, multicenter, blinded trials with longer follow up and targeted enrollment of patients with high baseline symptom burdens are warranted to fully characterize the clinical benefits of MCO dialyzers.

## Figures and Tables

**Table 1:** Dialyzer characteristics. Key specifications of the two dialyzers compared: Theranova 500 and Revaclear 500, including membrane type, inner fiber diameter, wall thickness, effective surface area, ultrafiltration coefficient, priming volume, and sterilization method.

Dialyzer Type	Dialyzer Membrane	Polymer Membrane Type	Inner Diameter (μm)	Wall Thickness (μm)	Effective Surface Area (m^2^)	Ultrafiltration Coefficient (mL/h/mmHg)	Priming Volume (mL)	Sterilization Method
Theranova 500	Medium cutoff	PAES/PVP, BPA-free	180	35	2	59	105	Steam
Revaclear 500	High flux	PAES/PVP, BPA-free	190	35	2.1	65	106	Steam

(Note: This table was reconstructed based on the original Hebrew data sheets. Exact manufacturer values should be cross verified).

**Table 2:** Baseline patient characteristics. Demographics, dialysis vintage, etiologies of ESRD, vascular access, and comorbidities for each treatment arm; values are mean ± SD or n (%), with between-group *p* values.

Baseline Characteristic	Theranova 500 (n = 20)	Revaclear 500 (n = 20)	*p* Value
Age, years (mean ± SD; range)	65 ± 14 (43–87)	69 ± 11 (54–84)	0.44
Hemodialysis vintage, months (mean ± SD; range)	55 ± 29 (32–87)	52 ± 29 (21–107)	0.66
ESRD cause (n,%)			0.26
Diabetic kidney disease	8 (40)	12 (60)	
Nephrosclerosis	4 (20)	2 (10)	
Cardiorenal syndrome	1 (5)	2 (10)	
Other/Unknown	7 (35)	4 (20)	
Dialysis access type (n,%)			0.36
AVF	13 (65)	15 (75)	
AVG	7 (35)	4 (20)	
Permcath	0 (0)	1 (5)	
Medical history (n,%)			0.06
Diabetes mellitus	8 (40)	12 (60)	
Hypertension	15 (75)	14 (70)	
Dyslipidemia	5 (25)	8 (40)	
Congestive heart failure	10 (50)	10 (50)	
Coronary artery disease	5 (25)	7 (35)	
Atrial fibrillation	9 (45)	12 (60)	

**Table 3:** Dialysis adequacy, dry weight, and ultrafiltration rates. spKt/V, prescribed dry weight, and delivered ultrafiltration volume at weeks 0, 12, and 24 for both groups; values are mean ± SD with *p* values for between-group comparisons.

	Theranova (n = 20)	Revaclear (n = 20)	*p* Value
spKt/V (mean ± SD)			
Week 0	1.24 ± 0.33	1.39 ± 0.32	0.213
Week 12	1.35 ± 0.27	1.44 ± 0.26	0.424
Week 24	1.4 ± 0.36	1.46 ± 0.27	0.645
Dry weight, Kg (mean ± SD)			
Week 0	75.31 ± 20.93	71.34 ± 13.3	0.536
Week 12	74.04 ± 17.85	72 ± 13.54	0.743
Week 24	73.75 ± 17.49	70.31 ± 13.85	0.598
UF, L (mean ± SD)			
Week 0	2.3 ± 1.48	2.39 ± 0.63	0.823
Week 12	2.11 ± 1.1	2.46 ± 0.71	0.328
Week 24	2.39 ± 1.28	2.23 ± 1.01	0.74

**Table 4:** Hemoglobin, iron stores, and mineral bone parameters. Serial measurements of hemoglobin, ferritin, transferrin saturation, serum calcium, phosphorus, and PTH at weeks 0, 12, and 24; values are mean ± SD with between-group *p* values.

	Theranova (n = 20)	Revaclear (n = 20)	*p* Value
Hemoglobin, g/dL (mean ± SD)			
Week 0	10.86 ± 1.2	10.86 ± 1.22	1
Week 12	11.04 ± 1.04	10.84 ± 0.86	0.6
Week 24	10.58 ± 1.15	11.53 ± 0.84	0.03
Ferritin, ng/mL (mean ± SD)			
Week 0	672.61 ± 540.33	480.47 ± 303.02	0.236
Week 12	574.31 ± 401.21	412.04 ± 239.83	0.215
Week 24	674.53 ± 415.86	681.62 ± 751.8	0.977
Transferrin saturation, % (mean ± SD			
Week 0	25.82 ± 10.64	30.36 ± 16.54	0.368
Week 12	28.19 ± 8.36	22.14 ± 8.65	0.084
Week 24	29.08 ± 12.52	25.2 ± 7.62	0.37
Calcium, mg/dL (mean ± SD)			
Week 0	8.49 ± 0.85	8.49 ± 0.62	0.983
Week 12	8.34 ± 1	8.59 ± 0.62	0.455
Week 24	8.17 ± 0.69	8.64 ± 0.78	0.128
Phosphorus, mg/dL (mean ± SD)			
Week 0	5.86 ± 1.54	6 ± 1.3	0.791
Week 12	6.66 ± 2.03	5.5 ± 0.62	0.053
Week 24	6.02 ± 1.58	5.71 ± 1.29	0.596
PTH, pg/mL (mean ± SD)			
Week 0	473.04 ± 484.91	415.45 ± 327.39	0.703
Week 12	703.28 ± 894.63	438.76 ± 464.86	0.343
Week 24	769.76 ± 1001.91	505.27 ± 598.37	0.441

## Data Availability

The data that support the findings of this study are not publicly available due to their containing information that could compromise the privacy of research participants, but are available from NLI the corresponding author.
